# A patient with pachydermoperiostosis harboring *SLCO2A1* variants with a history of differentiating from acromegaly

**DOI:** 10.1016/j.bonr.2023.101673

**Published:** 2023-03-16

**Authors:** Yukako Nakano, Yasuhisa Ohata, Makoto Fujiwara, Takuo Kubota, Yoko Miyoshi, Keiichi Ozono

**Affiliations:** a Department of Pediatrics, Osaka University Graduate School of Medicine, Suita 565-0871, Japan; b Faculty of Health and Nutrition, Osaka Shoin Women's University, Higashi-Osaka 577-8550, Japan

**Keywords:** Pachydermoperiostosis, Acromegaly, Hydrarthrosis, Prostaglandin transporter variant

## Abstract

Pachydermoperiostosis (PDP) is a rare hereditary disease characterized by digital clubbing, pachydermia, and periostosis. We describe a Japanese male patient with PDP who was differentially diagnosed with acromegaly by identification of compound heterozygous variants in *SLCO2A1*. Recent studies have reported various clinical manifestations, as well as skeletal and dermal features, in patients with PDP. Genetic testing provided not only PDP diagnosis and differentiation from acromegaly, but also information about possible complications and comorbidities throughout life.

## Highlights

• Genetic testing can differentiate PDP from other diseases with common symptoms.
• Not only dermal and skeletal symptoms but also anemia or gastrointestinal symptoms are observed in patients with PDP.
• Synovectomy was effective for joint symptoms and hydrarthrosis in this patient.

## 1 Introduction

Pachydermoperiostosis (PDP), also known as primary hypertrophic osteoarthropathy, is a rare monogenic disease characterized by three major symptoms: pachydermia, digital clubbing, and periostosis. Initially, Uppal et al. reported that pathogenic variants of the 15-hydroxyprostaglandin dehydrogenase (*HPGD*) gene caused PDP, after which, whole exome sequencing by Zhan et al. detected variants of the solute carrier organic anion transporter family member 2A1 (*SLCO2A1*) gene in patients with PDP (Uppal et al., 2008; Zhang et al., 2012). *HPGD* encodes 15-hydroxyprostaglandin dehydrogenase, which is thought to be a critical enzyme in the prostaglandin (PG) degradation pathway, whereby prostaglandin E2 (PGE2) is converted into PG metabolites (Uppal et al., 2008). *SLCO2A1* encodes a prostaglandin transporter protein that transports extracellular PGs to the cytoplasm for enzymatic inactivation (Schuster et al., 2015). Dysregulated expression of these genes results in the accumulation of PGE2 in the plasma and urine, which can be a useful diagnostic biomarker (Hou et al., 2018; Uppal et al., 2008; Zhang et al., 2012). Therefore, elevated plasma PGE2 levels are associated with PDP pathogenesis. To date, approximately 90 variants of *SLCO2A1* genes and 21 variants of *HPGD* genes have been reported worldwide (The Human Gene Mutation Database [HGMD], accessed July 2022). An updated study suggested that a monoallelic variant of *SLCO2A1* can cause mild PDP (Xu et al., 2021). PDP harboring *SLCO2A1*pathogenic variant is its preferable onset age of teenagers or early 20s, predominantly male and gastrointestinal signs and anemia as common complications. The case reported herein presented with gradually progressive digital clubbing, pachydermia, and periostosis, and recurrent hydrarthrosis, which served as an indication for considering PDP as a differential diagnosis.

## 2 Case

**Fig. 1:**
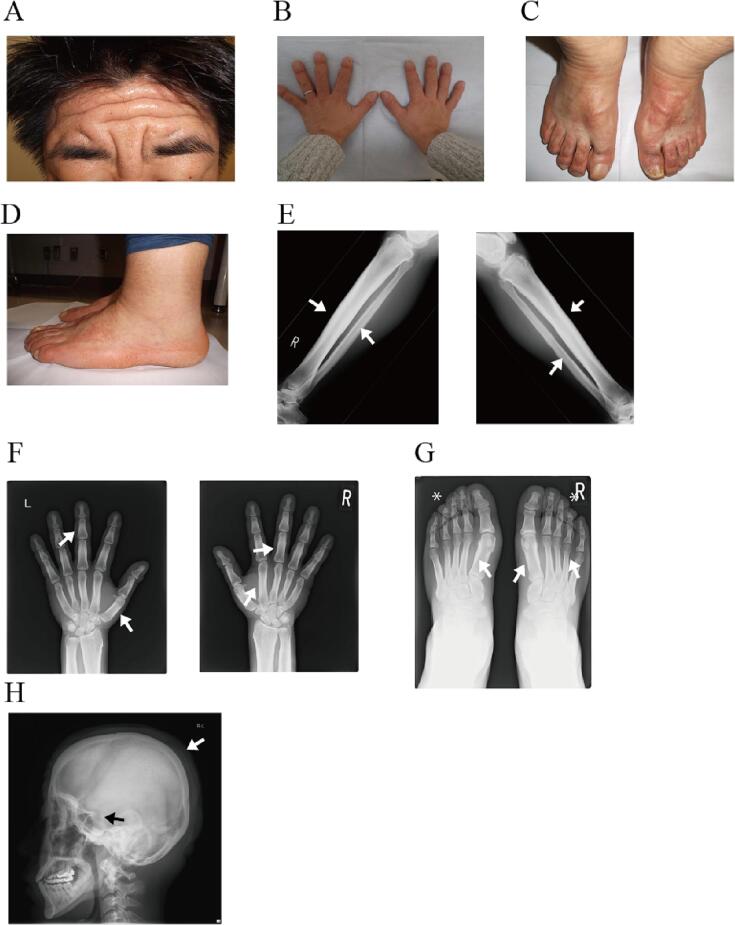
Clinical features of the proband at age 33. A. Thickened and corrugated skin of forehead, seborrhea, and hyperhidrosis. B. Skin thickening of hands and digital clubbing. C, D. Erythema in the top of the foot and cylindrical swelling of the legs. E. Bilateral symmetric periosteal thickening of the distal tibia and fibula (arrow). F. The white arrows denote the periosteal changes in some phalanges and metacarpals. G. The white arrows point the periosteal responses of some metatarsals. The asterisk indicates acro-osteolysis. H. Cortical and subperiosteal thickening in skull (white arrow); sella turcica is not enlarged (black arrow).

A 14-year-old boy presented to the orthopedic clinic with a six-month history of knee joint pain and impaired knee flexion. At the time of presentation, the patient was into skateboarding, and a diagnosis of Osgood-Schlatter disease was made. He refrained from sports and received non-steroidal anti-inflammatory drugs (NSAIDs) to relieve pain and inflammation. He subsequently presented with worsening knee pain, reduced knee flexion, and enlarged knee joints. The orthopedist recognized deformities of both knees and ankles, along with gradual dermal thickening of his swollen hands. At the age of 17 years, he was referred to the endocrinology department at the Osaka University Hospital for further investigation of acromegaly. On physical examination, his height and body weight were 179 cm (1.51 standard deviation [SD]) and 68 kg (0.57SD), respectively (SD was calculated based on SD adjusted by age and sex, Statistics Bureau Ministry of Internal Affairs and Communication 2018 retrieved from https://www.e-stat.go.jp/dbview?sid=0003224177). The heights of his father, mother and older sister were 168 cm, 168 cm and 160 cm, respectively. Serum level of insulin like growth factor 1 (IGF-1) was within normal range with respect to age and sex (47.5 nmol/L, average 40.0 nmol/L, −2.0 SD 18.6 nmol/L, 2.0 SD 70.6 nmol/L) (Isojima et al., 2012). Oral glucose tolerance test showed no excess growth hormone (GH) secretion (basal level was 0.15 μg/L and was suppressed to an undetectable level after loading). These observations suggested an unlikely diagnosis of acromegaly. Thereafter, he experienced recurrent knee hydrarthrosis, which forced him to limit exercise and required several intra-articular drainages. A radiograph of his knee showed thickening and fraying diaphysis in his lower extremities, without abnormality in the lumbar spine. This indicated a possibility of an underlying skeletal disease, for which he was referred to the orthopedic department at our hospital. Cortical thickening of the long bones was observed on radiography, accompanied by thickening of the hypertrophic hands, furrowing redundant skin on his forehead, and recurrent knee hydrarthrosis, leading to a diagnosis of PDP at the age of 20 years. Later, his reduced knee flexion had worsened over the years, resulting in synovectomy at age 22 to relieve his pain. The patient was free from knee hydrarthrosis after synovectomy. At the age of 33 years, he was referred to our department for confirmation of the PDP diagnosis and its hereditary concerns. He had an upper limit of height 182.5 cm (1.7 SD, average of 30s male 172.1 ± 6.1 cm) and average weight 80.5 kg (0.77 SD, average of 30s male; 71.0 ± 12.3 kg; Statistics Bureau Ministry of Internal Affairs and Communication 2018, retrieved from http://www.e-stat.go.jp/dbview?sid=0003224177). His heart rate and blood pressure were 83 beats/min and 122/58 mm Hg, respectively. He presented with excessive sebaceous secretions on his forehead and scalp, and thick, furrowed, and redundant skin on his forehead, with terminal enlargement of his fingers and cylindrical broadening of his ankles (Fig. 1A–D). Laboratory analyses revealed slightly elevated white blood cells count of 10,150/mm^3^ (reference range, 3300–9400/mm^3^), and the low vitamin D, which was 19.97 nmol/L (normal range > 49.92 nmol/L). Other laboratory profiles were unremarkable. On radiological examination, remarkable symmetrical periosteal changes were observed not only in the diaphysis of the fibula and tibia but also in the metacarpals, metatarsals, and proximal phalanx in both hands and feet (Fig. 1E–G). Acro-osteolysis affected the top of the distal phalanges in the feet but not in the hands (Fig. 1G). Thickening of the cortical region in the skull (Fig. 1H) was also manifested, with no abnormality in the spine. His knee swelling was apparent, with no joint pain or hydrarthrosis, and he had not taken any medication. He was born to non-consanguineous parents and his father had mildly enlarged hands. None of the other family members had these signs and symptoms.

### 2.1 Genetic analysis

**Fig. 2:**
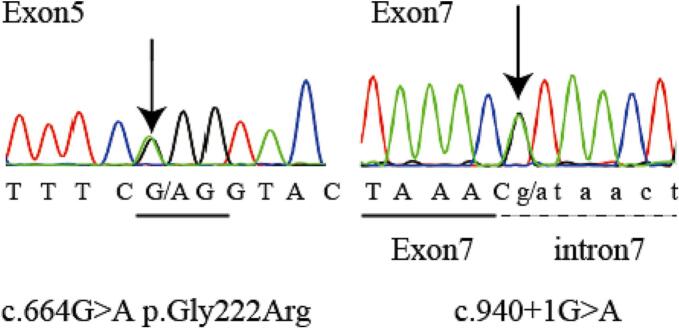
The location of the variants identified in the *SLCO2A1* gene in the proband (arrows).

Sequence analysis of the affected individual was performed by direct sequencing of the PCR products amplified from genomic DNA using the Big Dye Terminator Ready Reaction Kit, version 3.1 (Applied Biosystems, Foster City, California, USA). We examined all exons and exon-intron boundaries throughout the *SLCO2A1* and *HPGD* genes. We identified compound heterozygous variants in *SLCO2A1*, consisting of a heterozygous missense variant in exon 5 (c.664G>A, p.Gly222Arg) and a heterozygous splicing variant in intron 7 (c.940+1G>A; Fig. 2). These variants were previously identified and reported in another patient with PDP (Zhang et al., 2012). No variant in *HPGD* was detected. No material was available from his family members.

## 3 Discussion

We describe a Japanese man with a complete form of PDP, which was definitively diagnosed by genetic testing, in addition to his clinical presentations. His recurrent knee hydrarthrosis had led to a suspicion of PDP. Acromegaly is a common differential diagnosis in previously reported cases (Joshi et al., 2019; Karimova et al., 2017; Martinez-Lavin, 2011) and this diagnostic dilemma can be resolved if a remarkable elevation in growth hormone levels is observed. The radiographical features of PDP are also distinct from acromegaly, in which excessive soft tissue and/or enlarged joint space due to cartilage hypertrophy are observed (Woo, 1988), with another critical differential diagnosis being secondary hypertrophic osteoarthropathy (HOA). Since our patient had no respiratory and gastrointestinal symptoms, and no abnormality in his chest X-rays at his first consultation, we did not investigate the underlying diseases causing secondary HOA, especially malignancy. Over ten years had passed since the development of the first symptoms in the adolescent patient, and he has presented no other clinical signs and symptoms suggestive of any underlying diseases, which prompted no further investigation. He underwent an annual medical check-up including blood laboratory examination and chest radiography, as well as radiographic contrast study for upper gastrointestinal tract and abdominal ultrasonography once in several years. These analyses revealed a low limit of Hb level (Hb 129.0 g/L) and chronic gastritis at the age of 34.

Compound heterozygous variants in *SLCO2A1* consisting of a missense variant (c.664G>A, p.Gly222Arg) in exon 5 and a splicing variant (c.940+1G>A) in intron 7 were detected in the present case. These identical variants reported by Zhang Z et al. in 2012 resulted in a complete form of PDP in the patient, whose familial genetic testing revealed an autosomal recessive hereditary variant in his parents (Zhang et al., 2012). The missense variant (p.Gly222Arg) changes electric charges and helical structure, which is thought to result in impaired PG transporter function. The splicing variant (c.940+1G>A) impairs splicing, leading to the skipping of exon 7 and production of a frameshift and premature stop codon (p. Arg288Glyfs*7) (Zhang et al., 2012). The splicing variant has been identified in China, Japan, Korea, and European countries, and is thought to be the cause of an ancient founder effect in Japan (Lee et al., 2016; Sasaki et al., 2012). Although a number of case reports have been published worldwide, the prevalence of PDP is unknown owing to its rarity and lack of large-scale studies. Variants of *SLCO2A1* have been reported in Western and East Asian countries. According to recent research, diarrhea and anemia are commonly observed in PDP patients with *SLCO2A1* variants (Hou et al., 2018; Xu et al., 2021; Yuan et al., 2015; Zhang et al., 2013). Studies on phenotypic comparison between variants with *SLCO2A1* and *HPGD* showed that variants in *SLCO2A1* were predominantly detected in males, who presented with gastrointestinal symptoms, and high levels of serum and urinary prostaglandin E2 metabolites (PGEM); while a younger age-onset of PDP development in patients with *HPGD* variants was also reported (Hou et al., 2018; Yuan et al., 2015).

Some patients diagnosed with PDP develop Crohn's disease or myelofibrosis, and some patients with Crohn's disease or myelofibrosis also develop secondary HOA later in life (Compton et al., 1997; Diggle et al., 2012; Martinez-Lavin et al., 2008; Saghafi et al., 2008; Shim and Suh, 1997). Another report from Japan showed that some variants in *SLCO2A1* have been responsible for multiple chronic nonspecific ulcers of the small intestine, which is the same monoallelic variant our patient harbored (Umeno et al., 2015). These findings suggest that enteropathy is a possible symptom for PDP patients with the *SLCO2A1* variant, whereas only one patient with the *HPGD* variant has been reported to develop enteropathy to date (Yuan et al., 2015). The present case showed a lower limit of the normal range of Hb (133.0 g/L reference range 138.0 to 170.0 g/L), without any symptoms of chronic gastritis nor diarrhea. Patients with PDP present with a wide spectrum and varying degrees of symptoms. Therefore, genetic diagnosis and continuous monitoring will help clinicians detect potential complications early and improve their prognoses.

This patient took NSAIDs prior to a diagnosis of PDP, and his knee joint pain worsened. We could not obtain any information about the frequency, dose, and period of NSAIDs use. The development of recurrent hydrarthrosis appeared uncontrollable. Dermal symptoms and periostosis persisted, as shown in Fig. 1. In a review article investigating the effect of NSAIDs on symptoms observed in PDP, approximately 70 % of patients showed relief of arthritis or joint pain by NSAIDs, but the effect on dermal and skeletal lesions has not been described (Shakya et al., 2018). These observations suggest that NSAIDs have limited effects on joint symptoms.

This study has several limitations. We did not measure PGE2 and PGEM levels, which are biochemical hallmarks suggesting the diagnosis of PDP. However, the genetic test results provided a definite diagnosis. Another limitation is that familial genetic analyses were not available. Biallelic variants in *SLCO2A1* identified in our patient have been reported to be pathogenic in multiple studies, resulting in the conclusion of a causative variant in our patient (Zhang et al., 2012). Since some patients with PDP have been reported to develop Crohn's disease or myelofibrosis as late complications, long-term follow-ups and investigation for early detection are required.

In conclusion, we report the case of a Japanese man with a complete form of PDP harboring pathogenic compound heterozygous variants of *SLCO2A1*. Considering the pathogenic role of PGE2 in this disorder, a variety of systemic, skeletal, and dermal symptoms are expected to be observed in patients with PDP at any stage of their life. Therefore, genetic testing not only provides a definitive diagnosis, but is also recommended for better prognosis and treatment of possible complications and comorbidities.

## Conflict of interest

The authors declare no conflict of interest.

## Data Availability

Data will be made available on request.
